# Seasonal Variation in Flower Traits, Visitor Traits, and Reproductive Success of *Solanum sisymbriifolium* Lamarck (Solanaceae) in the Rarh Region of West Bengal, India

**DOI:** 10.3390/biology14070865

**Published:** 2025-07-16

**Authors:** Ujjwal Layek, Pappu Majhi, Alokesh Das, Prakash Karmakar, Arijit Kundu

**Affiliations:** 1 Department of Botany, Rampurhat College, Rampurhat 731224, West Bengal, India; layekujjwal@yahoo.co.in (U.L.); apualokesh@gmail.com (A.D.); 2 Department of Agricultural Engineering, Visva-Bharati, Sriniketan 731236, West Bengal, India; majhipappu97@gmail.com; 3 Department of Botany & Forestry, Vidyasagar University, Midnapore 721102, West Bengal, India; prakashbot1973@gmail.com

**Keywords:** buzz pollination, flower display size, flowering intensity, flowering phenology, pollination, pollination service index

## Abstract

The wild tomato (*Solanum sisymbriifolium*) is a globally distributed shrubby weed with both negative and positive impacts, including its invasive properties and the potential for pharmaceutical and traditional medicinal uses. Despite its ecological significance, the plant’s reproductive biology and pollination ecology remain poorly understood. This study aimed to investigate the floral biology, pollination ecology, and plant reproduction of the weed species. Some flower traits, such as flowering intensity, flower display size, and pollen and ovule production, peaked during spring, summer, and the monsoon, while flower longevity and stigmatic receptivity were the longest in winter. The plant species was self-compatible (ISI = 0.02), heavily depended on pollinators (IDP = 0.72), and experienced minimal pollination limitation (D = 0.10) under open-pollination conditions. Flower visitors’ traits (e.g., abundance, diversity, and richness) were higher in the spring, summer, and the monsoon, and these were lower in winter. The vital pollination service was provided by *Amegilla zonata*, *Ceratina binghami*, *Lasioglossum cavernifrons*, *Nomia* (*Curvinomia*) *strigata*, *Tetragonula pagdeni*, *Xylocopa aestuans*, *Xylocopa amethystina*, *Xylocopa fenestrata*, and *Xylocopa latipes*. Reproductive success, as indicated by fruit and seed set, varied seasonally, being higher during the spring–monsoon period and lower in winter. These findings support effective management of this weed species and help conserve the associated bee populations.

## Simple Summary

This study examined the seasonal variations in flower traits, plant–pollinator interactions, and reproductive success of *Solanum sisymbriifolium*. The flowering intensity, flower display size, and pollen and ovule production peaked during spring, summer, and the monsoon, while flower longevity and stigmatic receptivity were the longest in winter. The plant species showed self-compatibility and strong dependency on pollinators. Visitor traits also varied seasonally, with the peak during the spring–monsoon period. Vital pollinators were solitary bees and stingless bees. The fruit and seed sets were higher during the spring–monsoon period and lower in winter. This study will help in the management of the weed species and the conservation of associated bee populations.

## 1. Introduction

Flowering is a key stage in the life cycle of angiosperms, marking the shift from vegetative growth to reproductive maturity and playing a crucial role in the plant’s ability to produce seeds. This transition is foundational not only to individual survival and fecundity but also to broader population dynamics, species interactions, and ecosystem functioning [1,2]. Flowering influences ecological networks by determining the timing of resources, which affects pollinators, herbivores, pathogens, seed dispersers, and microbial interactions [3,4]. The characteristics of flowering—its onset, frequency, duration, and intensity—emerge from an intricate balance between internal regulators such as genetic programs and hormonal signals [5,6], and external stimuli including ambient temperature, day length, moisture availability, and soil fertility [7,8,9]. Across taxa and landscapes, these flowering patterns exhibit remarkable diversity, mirroring both evolutionary legacies and local adaptive responses. For example, Gentry [10] categorised the flowering patterns of Bignoniaceae into five types: steady state, modified steady state, cornucopia, big bang, and multiple bang. Frankie et al. [11] grouped flowering patterns into two categories: seasonal and extended, while Bawa [12] divided them into massive and extended types. These flowering patterns can influence the extent of outcrossing, availability of mates, frequency of near-neighbour matings, and overall reproductive output [13,14].

Floral characteristics lie at the heart of angiosperm reproductive ecology, shaping both the timing and success of pollination and fertilisation. Key attributes—including the flower longevity, moment of anthesis, the quantity of pollen and ovules produced, pollen viability, and the period of stigma receptivity—together govern a plant’s reproductive potential. These traits vary widely within and between species, influenced by environmental constraints and the evolution of mating systems [15,16]. For example, precise scheduling of flower opening can align pollen availability with peak pollinator foraging, while extended flower longevity may buffer against unpredictable pollinator visits. Likewise, the allocation of resources to pollen versus ovule production reflects strategic trade-offs in male and female function, and the overlap of pollen viability with stigma receptivity ensures optimal fertilisation.

A wide variety of animals visit the flowers of different plant species to collect resources like nectar and pollen, including insects such as bees, flies, butterflies, and beetles, as well as vertebrates like birds and bats. The species composition of the flower visitors’ spectrum is plant species-specific and also varies spatially and temporally [17,18]. The interaction between flowering plants and their animal visitors represents a cornerstone of terrestrial ecosystems, underpinning the reproductive success of most angiosperms. Not all floral visitors are effective pollinators; distinguishing between casual visitors and those that actively transfer pollen is key to understanding the ecological dynamics of plant reproduction. Accurately identifying which species are effective pollinators and understanding how their behaviours affect plant fitness are crucial for conserving biodiversity, sustaining ecosystem services, and ensuring the stability of plant–pollinator networks. The role of a pollinator is evaluated by measuring several parameters, including visitation pattern (legitimate or illegitimate), visitation rate, single-visit pollination efficiency [19], and pollinator importance [20]. As pollinator populations decline globally due to anthropogenic pressures such as habitat loss, pesticide use, and climate change [21,22], understanding the efficiency and specificity of these interactions becomes increasingly urgent.

Plant reproductive fitness, typically measured by fruit and seed set, serves as a vital indicator of a species’ capacity to sustain viable populations over time. In flowering plants, successful reproduction hinges on an intricate interplay of biotic elements, such as the presence and behaviour of pollinators, and abiotic factors, including temperature, rainfall, and photoperiod [9,23,24]. Seasonal fluctuations in these variables can significantly alter the timing, frequency, and efficiency of pollination [25,26], resulting in notable differences in both the quantity and quality of reproductive output. For instance, peak pollinator activity during optimal weather conditions may boost fruit set, while adverse conditions in off-peak seasons can suppress seed production or skew resource allocation.

Litchi tomato (*Solanum sisymbriifolium* Lam.) originates from South America and is a shrubby weed currently distributed worldwide, including hotter parts of India [27]. The plant exhibits various pharmaceutical activities, including antimicrobial and antioxidant properties [28,29]. Different plant parts have been widely used to treat numerous diseases, including diarrhoea, hypertension, and respiratory and urinary tract infections [30,31]. The plant is also used as a good trap crop against potato cyst nematodes, root-knot nematodes, and root-lesion nematodes [32]. Limited information exists on the floral and pollination biology of *Solanum sisymbriifolium*, as noted by Saha and Dutta [33] and Hill and Hulley [34]. Additionally, the seasonal variation in flower biology and reproductive ecology is not well understood. Plant–pollinator interactions are also not characterised for the plant species. Understanding the reproductive biology of a plant species is essential for effective population management. Therefore, a comprehensive study of the biology of the flower, plant–pollinator interactions, and reproduction of *Solanum sisymbriifolium* is crucial.

This study intended to expand knowledge on the reproductive biology of *Solanum sisymbriifolium* and assess the seasonal variation in flower traits, visitor traits, and plant reproductive success. Specifically, we investigated the following: (i) flower biology, (ii) floral visitors and pollinators, and (iii) the seasonal variation in flower traits, visitor traits, and plant reproductive success in the Rarh region of West Bengal, India. We hypothesised that flower traits change with the seasons, leading to fluctuations in plant–pollinator interactions and affecting the reproductive fitness of the species.

## 2. Materials and Methods

### 2.1. Study Area

We conducted surveys across various sites in the Rarh region (Bankura, Birbhum, and Paschim Medinipur districts) of West Bengal, India. The study areas characterise medium-density, mixed vegetation, with human habitats. The study area experiences seasonal variations—(i) summer (April to June, with daytime temperatures ranging between 35 and 42 °C), (ii) monsoon (July to August, day temperatures ranging 28 to 35 °C, an annual rainfall of 1479.9 mm), (iii) autumn (September to mid-October, day temperatures ranging between 30 and 33 °C), (iv) late autumn (mid-October to November, daytime temperature ranging from 29 to 32 °C), (v) winters (December to January, with daytime temperatures between 7 and 15 °C), and (vi) spring (February to March, daytime temperature ranging from 20 to 30 °C).

### 2.2. Plant Species

The study was conducted on *Solanum sisymbriifolium* Lam. (Solanaceae) from 2020 to 2023. The plant is known as vila-vila, sticky nightshade, or litchi tomato. It is originally native to Central and South America but is now found worldwide. The small, shrubby plant grows on roadside, uncultivated lands, and wastelands. The plant has sticky stems and branches with orange-yellow spines.

### 2.3. Floral Biology

The data about flower phenological events (e.g., flowering frequency, timing, and patterns) were recorded following the methods outlined by Gentry [10] and Hopkins [35]. We estimated flowering intensity (FI) and flower display size (FDS) as follows:

(1) Flowering intensity=Fn

(2) Flower display size=Ft

Here, *Fn* is the number of freshly open flowers (i.e., 1st day flowers) per day per reproductive unit, such as an inflorescence, flowering twig, or individual plant (in this case, an individual plant was considered). On the other hand, *Ft* is the number of total open flowers at a time per reproductive unit. For this purpose, individuals of old, medium, and young age classes (all of which had reached the reproductive phase) were chosen (N = 60 × 6 = 360 observations; 6 seasons, data equally derived from each age class). Flower display size was considered during the peak visitation period (i.e., 6:00–8:00 h) of flower visitors.

Flower opening and closing times were documented through field observations on flowers (N = 40 × 6 = 240 observations, 6 seasons) at a two-hour interval throughout the day, and the longevity of selected flowers was recorded.

Flower morphological traits were examined using various microscopes [including (i) a simple microscope, (ii) a compound light microscope (Primo Star, Zeiss), (iii) a stereo microscope (Stemi 508, Zeiss), and (iv) a field emission scanning electron microscope (FE-SEM, GeminiSEM 450, Gemini 2 column)]. The methods used for the SEM study are detailed in Table S1. For pollen count per flower, we collected all anthers of a mature flower bud (before dehiscence of anthers; N = 10 × 6 = 60 buds, 6 seasons) in a vial. We added 5 mL of sucrose solution to the anthers and crushed the anthers. Then, we added 1 mL of *Lycopodium* spore solution (which standardised about 75,600 spores/mL). Through microscopic observation, we counted *Lycopodium* spores and pollen grains from flowers. Then we estimated the number of flower pollens as ‘[(number of counted flower pollen ÷ number of counted *Lycopodium* spore) × number of *Lycopodium* spores added to solution]’ (as described by Layek et al. [36]). We studied pollen morphology (processed pollens: acetosyled according to Erdtman [37], and also unprocessed pollens) using a light microscope and a scanning electron microscope (detailed in Table S1). To count ovules per flower, we took an ovary (N = 40 × 6 = 240 ovaries, 6 seasons) on a glass slide, ruptured the ovary wall, and slightly pressed it to spread out the ovules. Then, we counted the ovules using a simple dissecting microscope.

Pollen viability was assessed using the 2,3,5-triphenyl tetrazolium chloride (TTC) staining method [38]. To prepare a 1% solution, 0.1 g of TTC and 6 g of sucrose were dissolved in 10 mL of distilled water. One drop of this solution was placed on a glass slide, and then pollen grains were added to the TTC solution. The pollen solution was veiled with a cover slip and kept in the dark for two hours. After this incubation period, a minimum of 100 pollen grains per sample were observed under a compound microscope. Grains exhibiting a reddish stain were classified as viable, whereas those displaying no colour change (or other colours like black or yellow) were considered non-viable. We tested pollen viability for all seasons (N = 10 × 6 = 60 flowers for pollen viability test, 6 seasons). Additionally, we assessed viability considering different day times with a 12 h interval starting from 0 h (i.e., during flower opening time) to 48 h after opening (N = 10 × 5 = 50 flowers, 5 time slots).

Pollen germinability was evaluated using an in vitro germination assay, following the protocol of Parfitt and Ganeshan [39], which utilised a germination medium consisting of 0.5% agar, 5 ppm boric acid, and 10% sucrose. A small quantity of the prepared medium was poured into sterile Petri dishes, and bulk pollen grains were then sprinkled onto the surface. The Petri dishes were incubated in the dark at 25 °C for 24 h. Following incubation, the medium containing pollen was gently transferred onto microscope slides for examination under a compound microscope. For each sample, at least 100 pollen grains were counted, and germination was considered successful when the length of the pollen tube was greater than the diameter of the corresponding pollen grain. Like the pollen viability test, the pollen germination test was conducted for each season (N = 10 × 6 = 60 flowers for the pollen germinability test, 6 seasons).

Stigma receptivity was assessed using the hydrogen peroxide test [40]. We collected flowers at different stages of development, spanning their longevity (e.g., 1st day morning, late afternoon; 2nd day morning, late afternoon; 3rd day morning, late afternoon, etc.), and tested stigma receptivity (*n* = 10 stigmas for each interval for each of the six seasons). For the last day of flower longevity, we tested receptivity at two-hour intervals, starting from morning until senescence. Stigmas, along with a small portion of the style, were carefully excised from the flowers using razor blades and placed on a glass slide. A drop of hydrogen peroxide (H_2_O_2_) solution was then applied, and after 10–15 s, stigma receptivity was examined under dissecting and stereo microscopes. The formation of numerous bubbles indicates receptivity to stigma, and a comparatively greater number of bubbles signifies a higher level of receptivity.

### 2.4. Mating System

To evaluate the mating system, we performed five pollination treatments during the peak flowering season in summer: (i) open pollination, (ii) pollinator exclusion to test for spontaneous autogamy, (iii) self-pollination (manually), (iv) cross-pollination (manually), and (v) supplementary pollination. In the late afternoon, matured flower buds were randomly selected (*n* = 100 flowers per treatment), marked, and enclosed in nylon mesh bags (except for those in the open and supplementary pollination treatments) to prevent external pollination until the floral parts were senesced. For manual self-pollination, we unbagged the flowers between 8:00 and 10:00 a.m., manually transferred pollen from the same flower to its stigma, and then immediately re-bagged the flowers. Flowers selected for cross-pollination treatment were emasculated prior to anther dehiscence. During the receptive period (8:00–10:00 h), the flowers were unbagged, pollens were added (collected from different individuals) on the stigma, and again re-bagged. In supplementary pollination, pollen (collected from the same or different individuals) was manually applied to the stigmas in addition to the natural pollination by native pollinators. Fruit development was recorded 5–7 days post-pollination, and seed set was assessed approximately 15 days thereafter for each treatment.

We calculated the index of self-incompatibility (ISI) using the method described by Raduski et al. [41], as follows:

(3) $$ISI=1-\frac{Rsp}{Rcp}$$

Rsp and Rcp refer to reproductive success in the self-pollination and cross-pollination treatments, respectively. We considered it as the seed set per flower. Based on the ISI value, a plant species can be classified into one of the three categories: (1) self-compatible (ISI ≤ 0.2), (2) partially self-incompatible (0.2 < ISI < 0.8), and (3) self-incompatible (ISI ≥ 0.8).

To determine the plant species’ dependency on pollinators, we calculated the index of dependency on pollinators (IDP) following the method described by Layek et al. [36], as outlined below:

(4) $$IDP=1-\frac{Re}{Rs}$$

Re and Rs represent the number of seeds produced per flower in the pollinator exclusion and supplementary pollination treatments, respectively. The IDP value ranges from 0 to 1, with higher values indicating a stronger dependence of the plant on pollinators.

To assess whether the plant species experience pollination limitations under open-field conditions, we calculated the pollination deficit coefficient (D) following the method described by Layek et al. [25], as detailed below:

(5) $$D=1-\frac{Ro}{Rs}$$

Ro represents the number of seeds produced per flower under open pollination. The value of D ranges from 0 to 1, with a higher value (D ≥ 0.1) indicating a significant pollination deficit in the plant species.

### 2.5. Floral Visitors

We conducted daytime field surveys to observe flower visitors, dividing the observation period into seven two-hour time slots between 4:00 and 18:00 h. Each survey, based on individual plant sampling, lasted 5 min per plant. One observation was conducted during each time slot per sampling day, totalling seven observations per day. Over the study period, a total of 1260 observations (N = 30 × 7 × 6 = 1260; 30 observations per time slot per season) were conducted. Visitors were either identified directly in the field or captured for identification at a later time.

Visitor abundance was estimated as the number of individuals observed per plant within a 5 min period. Next, we calculated the relative abundance (RA) for each flower-visiting species following Layek et al. [42]. Visitor richness was assessed using Margalef’s index (D) [43], and the diversity of flower visitors was measured using the Shannon-Weaver diversity index (*H′*) [44]. The methodologies are given in Table S2.

We recorded insect visitors collecting floral resources (e.g., nectar, pollen grains, and floral tissues). The flower visitation rate (VR), defined as the number of flowers visited per minute, was measured (*n* ≥ 20 observations for an insect species). For visitors with low visitation rates (such as beetles, flies, and stingless bees), the number of flowers visited over a 10 min period was recorded and then standardised to a per-minute rate. Additionally, flower handling time—the duration an insect spent on a single flower during a visit—was recorded (*n* ≥ 20 observations for an insect species).

### 2.6. Pollinating Strategies of Visitors

We recorded the visits made by different insect species on the flowers (N ≥ 300 visits). Then, we estimated flower visit proportion (FV) for each flower-visiting species as follows:

(6) $$FV=\frac{Vi}{Vt}$$

V*i* is the number of visits recorded for the *i* species, and V*t* is the total number of visits considering all species.

We recorded the visitors’ visitation patterns (i.e., legitimate and illegitimate types of visits). We examined the pollen adherence sites on visitors using a stereo microscope and a scanning electron microscope. For this purpose, visitors were captured using a medium-sized plastic container, immobilised by freezing, and then dried in a hot air oven (Digilab). Then, the samples were studied under a stereo microscope and a scanning electron microscope (the methodology is provided in Table S1).

For legitimate floral visitors, we documented various pollination modes (e.g., nototribic, sternotribic, notosternotribic, appendage-mediated, or a special type such as buzz pollination or pulsatory pollination [9]). To identify the vital pollinators of the plant species, we used a composite metric—the pollination service index (PS*i*)—by combining the standardised values (ranging from 0 to 1) of several key parameters.

(7) PSi=FV×FSi×AR×SR

FV is the flower visit proportion for visitor species *i*. FS*i* is the flower sex type selection index. For hermaphrodite flowers, each visitor is assigned an FS*i* value of 1. If a visitor’s FS*i* is greater than 1, all values are rescaled on a 0–1 scale, with the highest value normalised to 1 and the other values adjusted proportionally. AR and SR are the anther contact rate and stigma contact rates, respectively (calculated by dividing the number of visits involving contact by the total number of visits). When flower visit proportion (FV) data are unavailable, an alternative parameter can be used as a substitute [e.g., RA × VR/Σ(RA × VR); here RA is relative abundance and VR is flower visitation rate]. The pollination service index (PS*i*) ranges from 0 to 1, with higher values indicating a greater pollination service provided by the pollinator species.

### 2.7. Seasonal Reproductive Success

Fruit and seed sets were observed under open-field (i.e., unmanipulated) conditions throughout all seasons. In the morning, newly opened flowers (characterised by greenish-yellow anthers) were selected and marked by applying an ink spot to the pedicel and adding tags (*n* = 10 × 10 = 100 flowers per season, with ten sampling days per season). Subsequently, fruit and seed sets were recorded, following the same procedure as in the previously described pollination treatment experiment.

### 2.8. Statistical Analysis

We examined the data within each group, assessing key assumptions for parametric tests, including normality (using the Shapiro–Wilk test and Q-Q plots), homoscedasticity (using scatter plots and the Breusch-Pagan test), and the homogeneity of variance (using Levene’s test). The non-parametric Kruskal–Wallis H test was used for data that did not follow a normal distribution (e.g., seasonal flower traits: flowering intensity, flower longevity, ovule number, pollen production, pollen viability and germinability, stigma receptivity; seasonal visitor traits: abundance, richness, diversity; reproductive success: fruit and seed sets). When the *p*-value was significant (*p* ≤ 0.05), Dunn’s test was used as the post hoc analysis. Statistical analyses were conducted using IBM SPSS Statistics version 26.0 and R software 2022 (R Core Team, Vienna, Austria).

## 3. Results

### 3.1. Floral Biology

**Figure 1:**
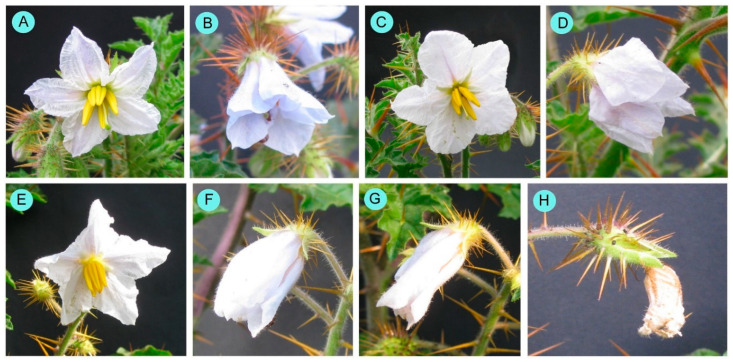
Flowers of different ages. (**A**) 1st day morning (freshly opened flower), (**B**) 1st day late afternoon, (**C**) 2nd day morning, (**D**) 2nd day late afternoon, (**E**) 3rd day morning, (**F**) 3rd day late afternoon, (**G**) 4th day morning, and (**H**) 5th day morning.

*Solanum sisymbriifolium* flowered consistently each year, displaying a steady-state flowering pattern. All individuals that had reached the reproductive stage bloomed synchronously. Flowering intensity showed significant seasonal variation (Kruskal–Wallis H test: χ^2^ = 39.12, df = 5, *p* < 0.001), with higher intensity observed in spring, summer, and the monsoon, and lower intensity during winter (Table 1). The flower display size also varied according to the seasons (Kruskal–Wallis H test: χ^2^ = 18.40, df = 5, *p* < 0.01), as did flowering intensity. The time of flower opening varied seasonally. During warmer seasons, such as summer and the monsoon, flowers opened earlier (between 5:00 and 6:00 a.m.) compared to the colder winter, when they opened later (between 7:00 and 8:00 a.m.). Flower longevity also varied across different seasons (Kruskal–Wallis H test: χ^2^ = 176.42, df = 5, *p* < 0.001). Comparatively, a higher flower longevity was observed during cold winter (55.60 ± 1.37 h) and lower in hot summer (32.60 ± 1.58 h). On the first day, in the late afternoon, the flowers became flaccid and closed up until the next morning. On the second day morning, closed flowers were reopened, and then closed again in the late afternoon (Figure 1). Anther colour also slightly changed from greenish yellow (in freshly opened flowers) to deep yellow in aged flowers.

**Figure 2:**
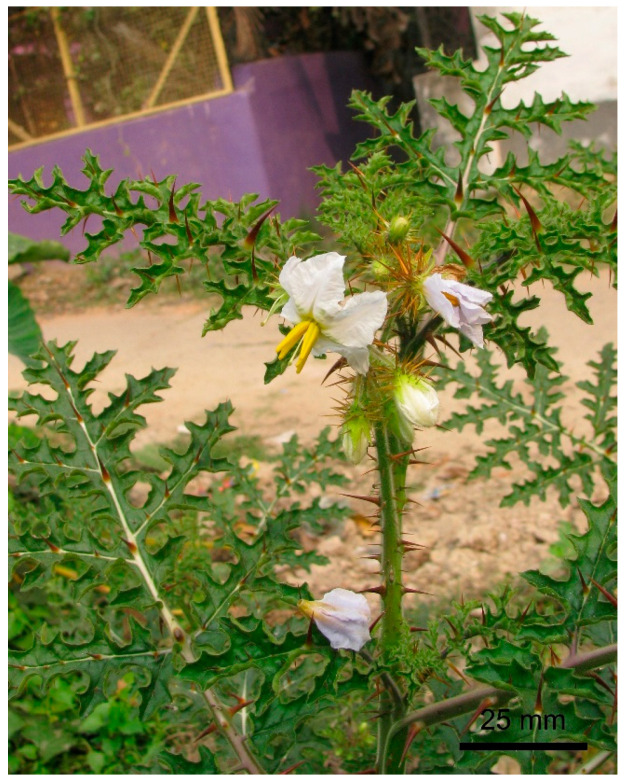
Flowering twig of *Solanum sisymbriifolium*.

**Figure 3:**
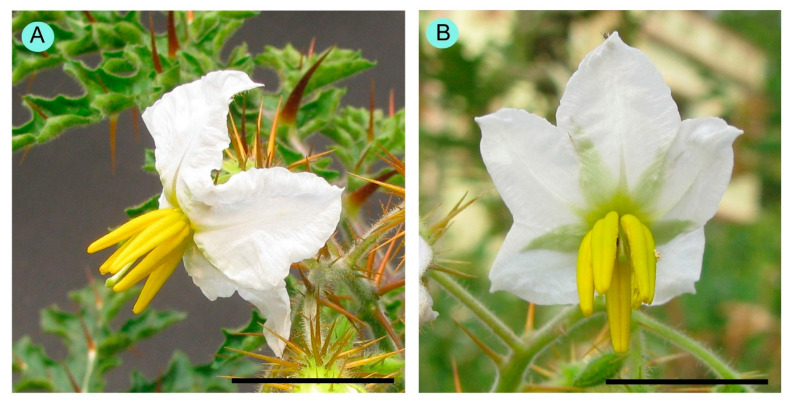
Two types of flowers of *Solanum sisymbriifolium*. (**A**) Fertile bisexual flower and (**B**) female-sterile flower. Scale bars = 20 mm.

**Figure 4:**
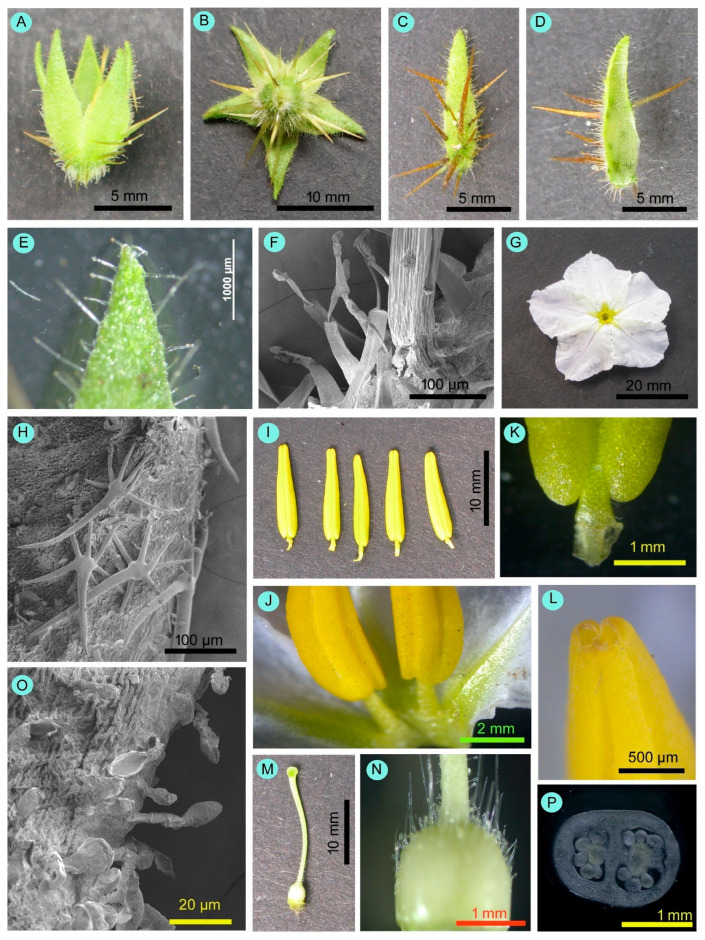
(**A**,**B**) Calyx; (**C**) outer surface of sepal; (**D**) inner surface of sepal; (**E**,**F**) a portion of sepal showing glandular hairs; (**G**) corolla; (**H**) outer surface of petal showing branched, star-shaped hairs; (**I**) androecium; (**J**) epipetalous stamens; (**K**) basifixed anther; (**L**) openings at the top of anther lobes; (**M**) gynoecium; (**N**) upper part of ovary showing hairs; (**O**) style surface showing glandular hairs; (**P**) t. s. of ovary showing axile placentation.

**Figure 5:**
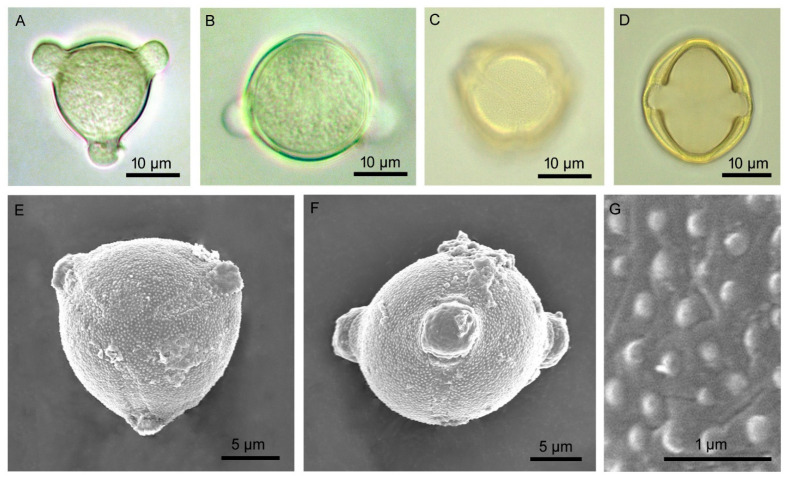
Microphotographs of pollen grains of *Solanum sisymbriifolium*. (**A**–**D**) Light microscopy (**A**,**B**: unprocessed pollens; **C**,**D**: processed pollens). (**E**–**G**) Scanning electron microscopy (**E**: polar view, **F**: equatorial view, **G**: exine ornamentation).

**Figure 6:**
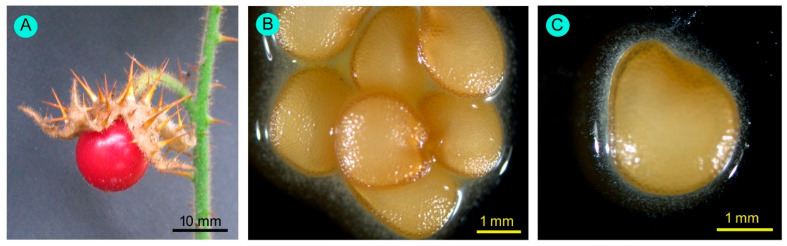
(**A**) Fruit and (**B**,**C**) seeds of *Solanum sisymbriifolium*.

Flowers were borne on raceme inflorescence (Figure 2). Inflorescences were 82.70 ± 34.78 mm (53.20–134.80 mm) in length. There were two types of flowers: (i) normal fertile bisexual flowers, and (ii) female-sterile flowers with short styles (Figure 3). Most flowers were normal, bisexual flowers, and female-sterile flowers were very rare (approximately 3% of the total flowers). Flowers were actinomorphic, bisexual, hypogynous, and white. The calyx had five sepals, which were gamosepalous (upper parts were free and fused at the base), persistent, and had a dorsal surface with spines of variable length and glandular hairs (Figure 4). The corolla had five petals, gamopetalous, with an acute apex, and was white. The outer surface of the petals bore branched, star-shaped hairs. Five stamens, epipetalous (attached at the base of petals), filaments very short (about 2.5 mm), anthers elongated (about 11 mm), basifixed, yellow-coloured, and dehisced by apical pores. The palynological analysis revealed that the pollen grains were monads with a spheroidal shape, measuring approximately 25.96 µm in diameter. Their ambs were sub-triangular, and they had trizonocolporate apertures. The exine of a pollen grain was about 2.5 µm thick and exhibited micro-verrucate ornamentation (Figure 5). Gynoecium had two carpels, syncarpous; stigma capitate, greenish, and apart from the anthers; style long (about 18 mm), whitish; ovary dome-shaped, and whitish. Many glandular hairs were on the style base and the upper part of the ovary. Ovules were many; placentation was the axile type. Fruit was berry type, globose, bright red when mature, and covered by an enlarged calyx. Each fruit contained many reniform seeds (Figure 6).

Each flower produced an average of 429,017.64 ± 86,608.11 pollen grains (mean ± SD, *n* = 60), while the number of ovules per flower averaged 74.08 ± 14.37 (mean ± SD, *n* = 240). The number of pollen grains and ovules per flower varied significantly across seasons (pollen grains: χ^2^ = 44.37, df = 5, *p* < 0.001; ovules: χ^2^ = 50.35, df = 5, *p* < 0.001). Pollen and ovule production were higher in the summer and monsoon seasons and lower during the cold winter (Table 1). The ovule to pollen ratio was 1:5791.27.

**Figure 7:**
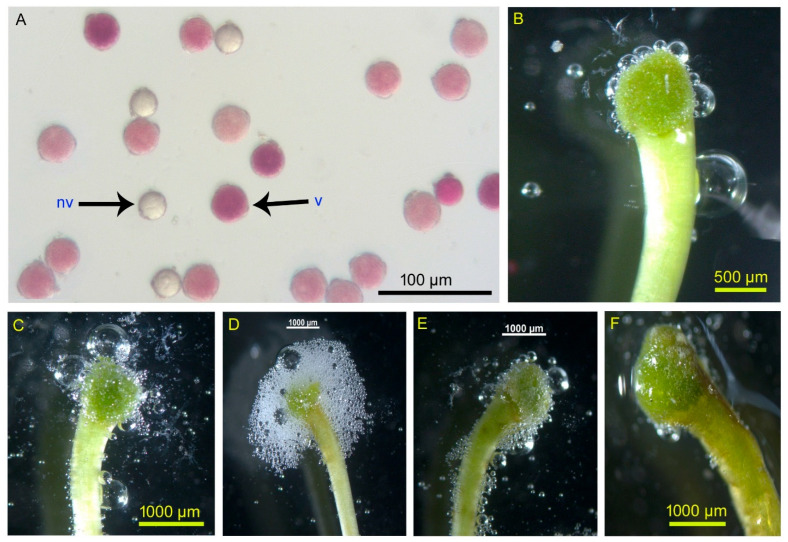
(**A**) Pollen viability test using the TTC staining method (v—viable, nv—non-viable), (**B**–**F**) stigma receptivity test using H_2_O_2_ (different ages of flowers: (**B**)—bud condition, (**C**)—1st day, (**D**)—2nd day, (**E**)—3rd day, (**F**)—4th day).

Anther dehiscence began 2 to 3 h after the flower had fully opened. Anther dehiscence was through apical pores (formation of a small opening at the top of the anther lobe). At the time of flower opening, pollen showed a viability of 82.16 ± 7.31% and a germinability of 76.82 ± 6.54%. Pollen viability and germinability showed no seasonal variation. However, pollen viability and germinability decreased with time elapsed (Table S3), being highest during opening time and very low by the 3rd day morning (i.e., at the end of flower longevity). Stigma remained receptive for the entire lifespan of the flower, from the moment it opened until it withered. Therefore, the flowers showed the protogynous type of dichogamy. During the time of flower opening (1st day flowers in the morning), stigma receptivity was lower, gradually reached its peak on the 2nd day flowers, and then decreased gradually. The duration of stigma receptivity varied significantly across seasons (χ^2^ = 83.74, df = 5, *p* < 0.001), being longest in winter (76.90 ± 0.23 h) and shortest in summer (51.80 ± 1.82 h). Figure 7 illustrates viable pollen and receptive stigmas.

### 3.2. Mating System

All five pollination treatments led to both fruit and seed formation (Table S4). Fruit and seed sets differed from pollinator exclusion treatments with other treatments (fruit set: χ^2^ = 27.01, df = 4, *p* < 0.001; seed set: χ^2^ = 145.54, df = 4, *p* < 0.001), with the lowest in pollinator exclusion treatment (fruit set: 45 ± 10.80%; seed set: 20.06 ± 23.87 seeds per flower). Fruit and seed sets did not differ among open pollination, manual selfing, manual crossing, and supplementary pollination treatments. The index of self-incompatibility (ISI) was calculated as 0.02, indicating that the plant species is fully self-compatible. The species exhibited a strong reliance on pollinators, with an index of dependency on pollinators (IDP) of 0.72. The coefficient of pollination deficit was very low (D = 0.10), suggesting that the plant species experienced minimal to no pollination limitation under open conditions.

### 3.3. Floral Visitors

**Figure 8:**
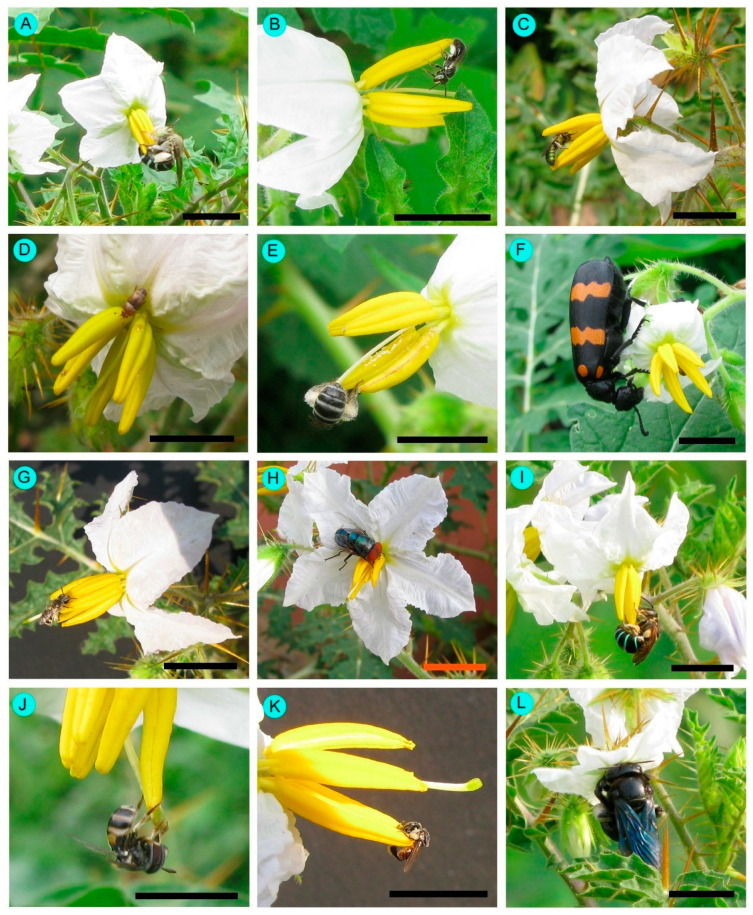
Flower visitors of *Solanum sisymbriifolium* in West Bengal. (**A**) *Amegilla zonata*, (**B**) *Brounsapis mixta*, (**C**) *Ceratina binghami*, (**D**) *Epuraea luteola*, (**E**) *Halictus* (*Seladonia*) *lucidipennis*, (**F**) *Hycleus phalarantha*, (**G**) *Lasioglossum cavernifrons*, (**H**) *Lucilia sericata*, (**I**) *Nomia* (*Curvinomia*) *strigata*, (**J**) *Paragus serratus*, (**K**) *Tetragonula pagdeni*, and (**L**) *Xylocopa fenestrata*. Scale bars = 10 mm.

A total of 21 insect species were recorded as floral visitors of *Solanum sisymbriifolium* in West Bengal, India (Table 2, Figure 8). The majority belonged to the order Hymenoptera, comprising 15 species. Within this group, most were from the family Apidae (12 species), followed by Halictidae (3 species).

The abundance, richness, and diversity of floral visitors fluctuated across seasons (Table 3). The greatest abundance, richness, and diversity of visitors were observed during summer (abundance: 2.40 ± 2.05 visitors/plant/5 min; richness, D = 0.52 ± 0.55; Shannon–Weaver diversity index, *H′* = 0.38 ± 0.38), while the lowest values occurred in winter (abundance: 0.88 ± 1.13 visitors/plant/5 min; richness, D = 0.23 ± 0.50; diversity index, *H′* = 0.13 ± 0.28). Visitor traits such as abundance, richness, and diversity showed significant variation across different times of the day (Table S5). The abundance, richness and diversity of flower visitors remained higher during 6.00–10.00 h for most seasons (except in winter, traits were higher at 10.00–12.00 h) and lower at 16.00–18.00 h.

The flower-visiting species with greater abundance were *Ceratina binghami* (relative abundance = 7.90%), *Epuraea luteola* (relative abundance = 16.21%), *Lasioglossum cavernifrons* (relative abundance = 14.63%), *Nomia* (*Curvinomia*) *strigata* (relative abundance = 13.60%), and *Tetragonula pagdeni* (relative abundance = 12.34%) (Table 2).

All the flower visitors collected pollen grains. Beetles also feed on floral tissues, especially the anther wall. The visitation rates for beetles and flies were very low. Among the hymenopteran members, the visitation rates were higher for *Amegilla zonata*, *Nomia* (*Curvinomia*) *strigata* and *Xylocopa* spp., while the visitation rate remained very low for stingless bees. Flower handling time was higher for beetles, flies, and stingless bees. The handling time for *Amegilla zonata* and *Xylocopa* spp. was very low.

**Figure 9:**
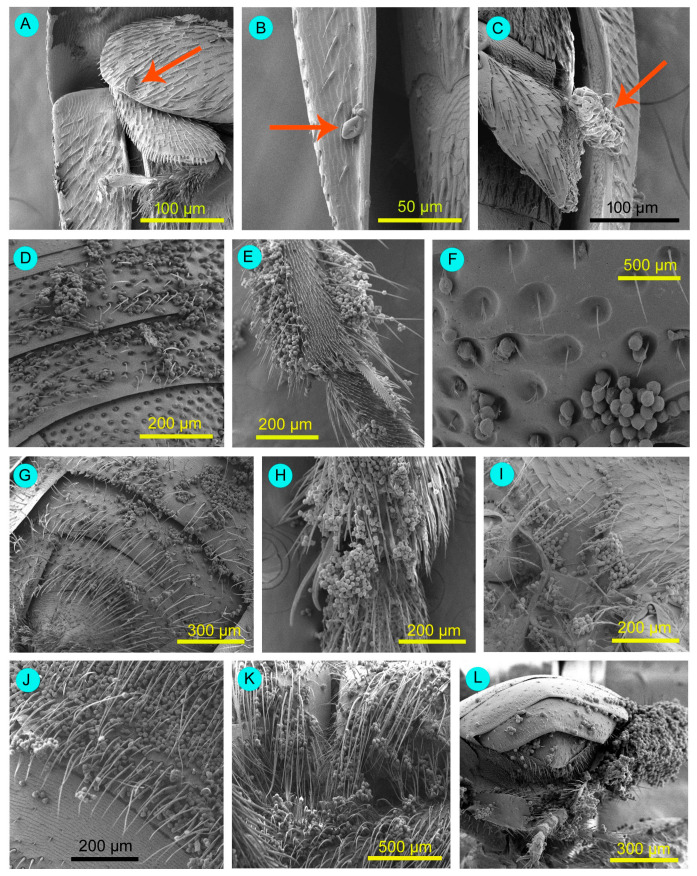
Showing attachment of pollen grains on insect body parts. (**A**–**C**) Illegitimate visitor, beetle *Epuraea luteola*. (**A**) Pollen attached to the segment of the leg, and (**B**,**C**) the margin of the wing. Arrows are showing attached pollen grains. (**D**–**L**) Legitimate visitors. (**D**–**F**) *Ceratina binghami* (ventral abdomen, leg, and ventral thorax, respectively), (**G**–**I**) *Lasioglossum cavernifrons* (ventral abdomen, leg, and ventral thorax, respectively), (**J**,**K**) *Nomia* (*Curvinomia*) *strigata* (ventral abdomen and ventral thorax), (**L**) *Tetragonula pagdeni* (pollen grains on legs and ventral abdomen and thorax).

The weed flowers received a higher amount of visits by *Nomia* (*Curvinomia*) *strigata* (FV = 0.224), *Lasioglossum cavernifrons* (FV = 0.143), *Amegilla zonata* (FV = 0.096), *Xylocopa fenestrata* (FV = 0.089), *Xylocopa aestuans* (FV = 0.088), *Tetragonula pagdeni* (FV = 0.075), and *Ceratina binghami* (FV = 0.068) (Table 2). The hymenopteran members (honeybees, solitary bees, and stingless bees) legitimately visited *Solanum sisymbriifolium* flowers (Table 2). Beetles and some flies visited flowers in an illegitimate manner. However, beetles and flies’ bodies adhered to *Solanum sisymbriifolium* pollens (Figure 9). Pollen was primarily attached to the legs—particularly the scopae in solitary bees and the corbiculae in honeybees and stingless bees—as well as to the ventral surfaces of the thorax and abdomen. Some solitary bees (e.g., *Amegilla zonata*, *Nomia* (*Curvinomia*) *strigata* and *Xylocopa* spp.) showed buzz pollination. They primarily contacted the stigma with the ventral side of their abdomen. Sometimes, they touched the stigmatic surface via the lateral sides of the thorax. Honeybees and stingless bees did not exhibit buzz pollination; instead, they employed the sternotribic pollination mode via the ventral surface of their abdomen and thorax. Bees also conducted appendage-mediated pollination. The anther touching rates were higher for all bee species. Stigma touching rates were higher for blue banded bees (*Amegilla zonata*) and carpenter bees (*Xylocopa* spp.). Based on the pollination service index (PS*i*), vital pollinators were *Nomia* (*Curvinomia*) *strigata* (PS*i* = 0.116), *Amegilla zonata* (PS*i* = 0.088), *Xylocopa fenestrata* (PS*i* = 0.085), *Xylocopa aestuans* (PS*i* = 0.083), *Lasioglossum cavernifrons* (PS*i* = 0.069), *Xylocopa latipes* (PS*i* = 0.033), *Ceratina binghami* (PS*i* = 0.029), and *Tetragonula pagdeni* (PS*i* = 0.028).

### 3.4. Seasonal Plant Reproduction

**Figure 10:**
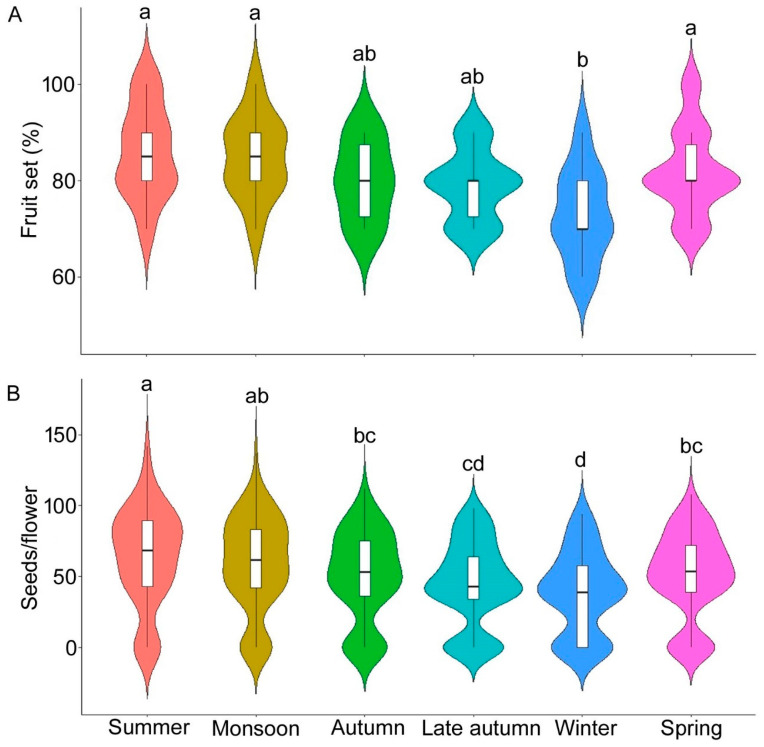
Season-wise fruit set (**A**) and seed set (**B**) of *Solanum sisymbriifolium* in West Bengal, India. Different superscript letters within a row (i.e., season-wise) indicate significant differences (Kruskal–Wallis H test and Dunn’s post hoc test at 0.05% level).

Reproductive success, measured by fruit and seed set, varied significantly across seasons (fruit set: χ^2^ = 11.19, df = 5, *p* < 0.05; seed set: χ^2^ = 46.22, df = 5, *p* < 0.001). The fruit set remained higher during spring (82 ± 9.19%), summer (86 ± 9.66%) and monsoon (85 ± 8.50%) (Figure 10). Seed set was the highest during summer (63.86 ± 34.20 seeds/flower) and the lowest in winter (38.68 ± 28.59 seeds/flower). During summer, the plant produced a higher number of seeds (63.86 ± 34.20 seeds per flower), while seed set was lower in winter (38.68 ± 28.59 seeds per flower).

## 4. Discussion

The classification of flowering patterns in angiosperms has been conducted in various ways by several researchers. For example, Gentry [10] mentioned five types of flowering patterns (cornucopia, big bang, multiple bang, steady state, and modified steady state), Bawa [12] divided into two groups (i.e., extended and massive), and Frankie et al. [11] classified flowering patterns into two groups (i.e., extended and seasonal). The plant species under study displayed steady-state flowering year-round in the Rarh region of West Bengal. A similar flowering pattern has been observed in many tropical plant species (e.g., *Turnera ulmifolia* [9]). Steady-state flowering enhances cross-pollination and may ensure reproductive success under adverse weather. Synchronous blooming among individuals allows widespread gene exchange within the population, boosting genetic diversity [45,46]. The majority of flower traits, including flowering intensity, flower longevity, ovule number, pollen production, and duration of stigma receptivity, varied seasonally in Rarh Bengal. This may be due to variations in seasonal atmospheric factors. The responsiveness of floral traits to environmental factors has been well documented in many plant species (e.g., *Hordeum vulgare* [47], *Theobroma cacao* [48], and *Turnera ulmifolia* [9]). Certain floral traits—such as flowering intensity, flower display size, and ovule and pollen production—were enhanced during the summer and monsoon season and declined in winter. In contrast, some others (e.g., flower longevity and duration of stigma receptivity) showed a reverse trend. In winter, an extended period of stigma receptivity may help offset the low flowering frequency, thereby improving reproductive success [49]. This trait likely aids biotic pollination under harsh conditions. The duration of female receptivity is also influenced by the occurrence of pollination [50,51], and long-lasting stigmas increase the chances of cross-pollination. The pollen viability and germinability did not vary seasonally but significantly decreased with the ageing of flowers. During senescence (especially 3rd day old flowers), viability and germinability were retained very little.

In terms of anther and stigma maturation, the plant species exhibited protogyny, a condition less common in angiosperms compared to protandry. It has also been reported for some other Solanaceae members, such as *Anthocercis gracilis* [52], *Jaltomata sinuosa* [53], and *Mandragora caulescens* [54]. This form of dichogamy is highly effective in promoting outcrossing in the species. Based on its mating system, fruit and seed development in bagged flowers suggested that the plant species is capable of spontaneous self-pollination. This strategy provides reproductive assurance in challenging environments where pollinator activity is limited. Additionally, similar seed set levels between self-pollination and cross-pollination treatments suggested that the species was self-compatible. Among different species of *Solanum*, researchers found both phenomena: self-compatibility [55,56] and self-incompatibility [52,57,58]. The reproductive assurance is considered a major selective force driving the evolution of self-fertilisation [59,60]. Self-fertilisation enables individuals to reproduce even when the success of cross-fertilisation is constrained by limited access to compatible mates or pollinators. Additionally, selfing may enhance the colonisation ability of a plant species [61]. In contrast, cross-fertilisation is thought to be superior to selfing, considering it reduces inbreeding, which is often associated with the production of offspring of lower genetic quality [62]. Seed set in the pollinator exclusion treatment was relatively lower than in the supplementary pollination treatments, indicating that the plant species has a moderate reliance on biotic pollinators. The pollinator dependence of fruit and seed sets has also been reported for various species of *Solanum* (e.g., *Solanum aethiopicum* [63], *Solanum anguivi* [63], and *Solanum melongena* [64]). In open field conditions, the plant species had a negligible or very low pollination deficit (coefficient of pollination deficit, D = 0.10). Pollination limitation in a plant species is influenced by floral traits, pollinator availability, and weather conditions during the flowering period [9,36,65].

Only a limited amount of work has been performed on the flower visitors of the weed species. Saha and Dutta [33] worked from Tripura, India, and Hill and Hulley [34] reported flower visitors for the weed from South Africa. We provided detailed information about flower visitors and their interactions on *Solanum sisymbriifolium*, for the first time from the Rarh region of West Bengal, India. The weed flowers were visited by beetles, flies, honeybees, solitary bees, and stingless bees, with beetles, solitary bees, and stingless bees being the most dominant. The most solitary bees (e.g., *Amegilla zonata*, *Nomia* (*Curvinomia*) *strigata*, *Xylocopa* spp.) showed buzzing activity on the weed species. The buzzing activity of visitors is well established on the Solanaceae members [66,67]. The effective pollinators (based on pollination service index, PS*i*) of the weed species were solitary bees (e.g., *Amegilla zonata*, *Lasioglossum cavernifrons*, *Nomia* (*Curvinomia*) *strigata*, *Xylocopa* spp.) and stingless bees (*Tetragonula pagdeni*). Previous studies (e.g., Saha and Dutta [33]; Hill and Hulley [34]) have also shown the importance of blue-banded bees (*Amegilla* spp.) and carpenter bees (*Xylocopa* spp.) as pollinators of the weed. However, the composition of insect species varied across studies. The composition of flower visitors depends not only on the plant species but also varies across time and space [18]. Regarding temporal variation, visitor traits, including abundance, richness, and diversity, were higher in summer and lower in winter. The greater flowering intensity and flower display size during summer positively influenced visitor traits. The influence of floral traits on pollinator characteristics has been demonstrated in several plant species by numerous researchers (e.g., McCall and Primack [68]; Ebeling et al. [69]; Layek et al. [9]). The pollinator traits are also influenced by the surrounding vegetation [36,70], as it deters the availability of food and habitat for native pollinators.

The fruit and seed set of the weed species showed seasonal variation, with higher levels observed in spring, summer, and the monsoon season, and lower levels during winter. Reproductive success (i.e., fruit and seed set) of a plant species depends on several factors, including nutrient availability [71], the physiological condition of the individuals [72], and pollination services [36,73]. The pollination service is also linked to flowering intensity, pollinator traits and weather conditions [9,74]. During the spring to monsoon season, the higher flowering intensity and floral display size of the weed species remained more attractive to pollinators, resulting in higher pollinator abundance, increased pollination services, and ultimately, higher reproductive success. Additionally, flower traits, especially a higher number of ovule production during these seasons, were also associated with higher seed set. A positive correlation between ovule production and seed set has been well documented by several researchers (e.g., Strelin and Aizen [75]; Layek et al. [9]).

## 5. Conclusions

The wild tomato (*Solanum sisymbriifolium*) bloomed throughout the year with a steady-state pattern. Flower traits varied seasonally, with some (e.g., flowering intensity, flower display size, ovule and pollen production) peaking during spring, summer, and the monsoon, while values remained lowest during winter. Some other flower traits, such as flower longevity and the duration of stigma receptivity, exhibited a reverse seasonal variation. The plant species exhibited full self-compatibility (ISI = 0.02), showed a high reliance on pollinators (IDP = 0.72), and faced only slight pollination limitation under open conditions (pollination deficit coefficient, D = 0.10). Considering flower visitors, beetles, flies, honeybees, solitary bees, and stingless bees visited *Solanum sisymbriifolium* flowers. The most abundant visitors were *Epuraea luteola*, *Lasioglossum cavernifrons*, *Nomia* (*Curvinomia*) *strigata*, and *Tetragonula pagdeni*. Based on the pollination service index (PS*i*), effective pollinators were *Amegilla zonata*, *Ceratina binghami*, *Lasioglossum cavernifrons*, *Nomia* (*Curvinomia*) *strigata*, *Tetragonula pagdeni*, *Xylocopa aestuans*, *Xylocopa amethystina*, *Xylocopa fenestrata*, and *Xylocopa latipes*. The reproductive success of the plant, as measured by fruit and seed set, varied seasonally. The fruit and seed set remained higher during hot seasons (i.e., spring–monsoon) and comparatively lower in cold winter. The present work provided information about flower biology, visitors and plant–pollinator interactions on the weed species, and these data will help in the management of the weed and also in the conservation of the associated wild bee fauna.

## Figures and Tables

**Table 1:** Flower traits of *Solanum sisymbriifolium* across different seasons in West Bengal, India.

Flower Traits	Summer	Monsoon	Autumn	Late Autumn	Winter	Spring	Statistics
Flowering intensity	3.85 ^a^ ± 4.61	4.12 ^a^ ± 5.12	3.03 ^ab^ ± 2.97	2.13 ^bc^ ± 1.98	1.33 ^c^ ± 1.46	3.47 ^a^ ± 2.49	χ^2^ = 39.12, ***
Flower display size	7.75 ^a^ ± 9.21	8.18 ^a^ ± 9.73	6.47 ^ab^ ± 6.14	5.68 ^bc^ ± 5.17	4.13 ^c^ ± 4.58	7.17 ^a^ ± 4.81	χ^2^ = 18.40, ***
Flower longevity (hours)	32.60 ^d^ ± 1.58	33.10 ^d^ ± 1.81	33.90 ^d^ ± 1.92	48.05 ^b^ ± 8.98	55.60 ^a^ ± 1.37	38.10 ^c^ ± 6.55	χ^2^ = 176.42, ***
Pollen (no. of grains flower^−1^)	515,306.93 ^a^ ± 49,153.55	520,167.56 ^a^ ± 21,340.97	428,793.87 ^b^ ± 60,018.43	350,910.06 ^c^ ± 32,606.08	319,852.16 ^c^ ± 11,734.98	439,075.23 ^b^ ± 62,140.65	χ^2^ =44.37, ***
Ovule (no. of ovules flower^−1^)	80.25 ^a^ ± 15.01	83.20 ^a^ ± 13.97	73.38 ^b^ ± 13.40	68.32 ^bc^ ± 11.57	64.95 ^c^ ± 11.40	74.38 ^b^ ± 12.52	χ^2^ = 50.35, ***
Pollen viability	79.98 ± 7.23	80.73 ± 7.02	81.05 ± 7.11	81.77 ± 7.38	82.16 ± 7.31	81.54 ± 7.28	χ^2^ = 1.25, *p* = 0.94
Pollen germinability	74.35 ± 6.42	74.76 ± 6.25	75.12 ± 6.32	75.86 ± 6.57	76.82 ± 6.54	75.27 ± 6.51	χ^2^ = 1.23, *p* = 0.82
Stigma receptivity (duration in hours)	51.80 ^c^ ± 1.82	52.40 ^c^ ± 1.90	52.90 ^c^ ± 1.89	57.50 ^b^ ± 5.76	76.90 ^a^ ± 2.29	56.30 ^b^ ± 4.51	χ^2^ = 83.74, ***

Values are presented as mean ± standard deviation. Different superscript letters within a row (i.e., across seasons) indicate statistically significant differences (Dunn’s post hoc test at the 0.05 level). Statistics: df = 5 for each parameter, ***—*p* < 0.001.

**Table 2:** Flower visitors of *Solanum sisymbriifolium* in West Bengal, India.

Flower Visitor	RA	VR	HT	Resource	Pollination Strategies							
					FV	Visitation Pattern	Buzzing Activity	Pollinating Mode	Pollen Adhering Parts	AR	SR	PS*i*
Coleoptera												
*Epuraea luteola*	16.21	0.11 ± 0.03	-	P	0.011	IL	No	-	H, VA, VT, L	-	-	-
*Hycleus phalarantha*	0.19	0.10 ± 0.0	-	FT	0.001	IL	No	-	-	-	-	-
Diptera												
*Eristalinus megacephalus*	0.51	0.34 ± 0.13	-	P	0.003	IL	No	-	H, L	-	-	-
*Helophilus peregrinus*	0.33	0.32 ± 0.14	-	P	0.002	IL	No	-	H, L	-	-	-
*Lucilia sericata*	1.17	0.28 ± 0.10	-	P	0.002	IL	No	-	H, L	-	-	-
*Paragus serratus*	1.03	1.40 ± 0.57	-	P	0.003	L	No	-	H, L, VA, VT	1	0.53	0.002
Hymenoptera												
*Amegilla zonata*	6.45	5.13 ± 1.20	0.74 ± 0.23	P	0.096	L	Yes	S, A	VA, VT, L	1	0.92	0.088
*Apis cerana*	1.31	3.43 ± 1.20	15.54 ± 22.17	P	0.014	L	No	S, A	VA, VT, L	1	0.44	0.006
*Apis dorsata*	1.45	3.73 ± 1.38	14.38 ± 20.26	P	0.016	L	No	S, A	VA, VT, L	1	0.45	0.007
*Apis florea*	0.56	3.27 ± 1.07	16.63 ± 21.75	P	0.005	L	No	S, A	VA, VT, L	1	0.42	0.002
*Brounsapis mixta*	3.69	2.43 ± 1.13	12.04 ± 13.38	P	0.029	L	No	S, A	VA, VT, L	1	0.40	0.012
*Ceratina binghami*	7.90	2.87 ± 1.21	20.52 ± 29.81	P	0.068	L	No	S, A	VA, VT, L	1	0.42	0.029
*Ceratina hieroglyphica*	2.43	3.51 ± 1.49	15.85 ± 24.64	P	0.026	L	No	S, A	VA, VT, L	1	0.41	0.011
*Halictus* (*Seladonia*) *lucidipennis*	3.36	3.33 ± 1.47	15.73 ± 23.80	P	0.024	L	No	S, A	VA, VT, L	1	0.41	0.010
*Lasioglossum cavernifrons*	14.63	3.17 ± 1.39	17.68 ± 28.44	P	0.143	L	Yes	S, A	VA, VT, L	1	0.48	0.069
*Nomia (Curvinomia) strigata*	13.60	5.97 ± 1.71	8.18 ± 10.23	P	0.224	L	Yes	S, A	VA, VT, L	1	0.52	0.116
*Tetragonula pagdeni*	12.34	0.74 ± 0.26	100.78 ± 82.15	P	0.075	L	No	S, A	VA, VT, L	1	0.37	0.028
*Xylocopa aestuans*	4.49	6.53 ± 1.53	1.38 ± 0.50	P	0.088	L	Yes	S, A	VA, VT, L	1	0.94	0.083
*Xylocopa amethystina*	2.10	7.27 ± 1.64	0.78 ± 0.24	P	0.045	L	Yes	S, A	VA, VT, L	1	0.91	0.041
*Xylocopa fenestrata*	4.30	6.97 ± 1.38	1.29 ± 0.48	P	0.089	L	Yes	S, A	VA, VT, L	1	0.96	0.085
*Xylocopa latipes*	1.96	6.10 ± 1.63	1.42 ± 0.53	P	0.036	L	Yes	S, A	VA, VT, L	1	0.92	0.033

RA—relative abundance (%); VR—flower visitation rate; HT—flower handling time (s); Resource: FT—floral tissue, P—pollen; FV—flower visit proportion; Visitation pattern: IL—illegitimate, L—legitimate; Pollinating mode: S—sternotribic, A—appendage-mediated; Pollen adhering body parts: H—head, L—legs, VA—ventral side of abdomen, VT—ventral side of thorax; AR—anther contacting rate; SR—stigma contacting rate; PS*i*—pollination service index.

**Table 3:** Abundance, richness, and diversity of floral visitors on *Solanum sisymbriifolium* in West Bengal.

Season	Abundance	Richness	Diversity
Summer	2.40 ^a^ ± 2.05	0.52 ^a^ ± 0.55	0.38 ^a^ ± 0.38
Monsoon	2.07 ^ab^ ± 2.05	0.48 ^ab^ ± 0.55	0.34 ^ab^ ± 0.39
Autumn	1.60 ^cd^ ± 1.71	0.39 ^bc^ ± 0.56	0.26 ^cd^ ± 0.36
Late autumn	1.33 ^d^ ± 1.41	0.30 ^cd^ ± 0.50	0.19 ^de^ ± 0.31
Winter	0.88 ^e^ ± 1.13	0.23 ^e^ ± 0.50	0.13 ^e^ ± 0.28
Spring	1.91 ^bc^ ± 1.83	0.43 ^ab^ ± 0.54	0.30 ^bc^ ± 0.38
Throughout year	1.70 ± 1.80	0.39 ± 0.54	0.27 ± 0.36
Statistical analysis	χ^2^ = 87.45, df = 5, *p* < 0.001	χ^2^ = 51.72, df = 5, *p* < 0.001	χ^2^ = 62.99, df = 5, *p* < 0.001

Abundance: number of visitors/individual/5 min; richness: Margalef’s index D; diversity: index of Shannon-Weaver, *H’*; Data are presented as mean ± standard deviation. Different superscript letters within the same column denote statistically significant differences (Dunn’s post hoc test, *p* < 0.05).

## Data Availability

Data were available within the article and Supplementary Files.

## Supplementary Materials

The following supporting information can be downloaded at: https://www.mdpi.com/article/10.3390/biology14070865/s1. Table S1. The methodologies for the microscopic study of flower parts and pollen grains; Table S2. Methodologies for determining relative abundance, richness and diversity of flower visitors; Table S3. Flower-age-wise pollen viability and germinability of *Solanum sisymbriifolium* in West Bengal, India; Table S4. Fruit and seed sets in different pollination treatments on *Solanum sisymbriifolium* in West Bengal, India; Table S5. Daytime-wise, visitor traits (abundance, richness, and diversity) of *Solanum sisymbriifolium* in West Bengal, India.
