# Supplementary material for: Seasonal Variation in Flower Traits, Visitor Traits, and Reproductive Success of Solanum sisymbriifolium Lamarck (Solanaceae) in the Rarh Region of West Bengal, India
Source: Biology (Basel). 2025 Jul 16;14(7):865. doi: 10.3390/biology14070865 (PMC12292435; doi:10.3390/biology14070865)
Supplement: Supplementary file 1 [file biology-14-00865-s001.zip › 16. Table S3.pdf]

Table S3. Flower-age-wise pollen viability and germinability of *Solanum sisymbriifolium* in West Bengal, India.

**Parameter**	**Flower age from opening time**				
	0 hr.	12 hrs.	24 hrs.	36 hrs.	48 hrs.
Pollen viability (%)	82.18 ± 7.31	76.32 ± 7.24	69.89 ± 7.18	56.49 ± 6.53	40.28 ± 5.87
Pollen germinability (%)	76.82 ± 6.54	71.27 ± 6.42	64.60 ± 6.21	51.44 ± 6.08	36.35 ± 5.80

Values are given in mean ± standard deviation.
