# Supplementary material for: Seasonal Variation in Flower Traits, Visitor Traits, and Reproductive Success of Solanum sisymbriifolium Lamarck (Solanaceae) in the Rarh Region of West Bengal, India
Source: Biology (Basel). 2025 Jul 16;14(7):865. doi: 10.3390/biology14070865 (PMC12292435; doi:10.3390/biology14070865)
Supplement: Supplementary file 1 [file biology-14-00865-s001.zip › 17. Table S4.pdf]

Table S4. Fruit and seed sets in different pollination treatments on *Solanum sisymbriifolium* in West Bengal, India.

Pollination treatment	Fruit set (%)	Seed set (seeds/flower)
Open pollination	$86^{a} \pm 9.66$	$63.86^{a} \pm 34.20$
Pollinator exclusion	$45^{b} \pm 10.80$	$20.06^{b} \pm 23.87$
Manual selfing	$90^{a} \pm 8.16$	$68.35^{a} \pm 31.12$
Manual crossing	$92^{a} \pm 7.89$	$69.98^{a} \pm 29.83$
Supplementary pollination	$93^{a} \pm 8.23$	$71.28^{a} \pm 29.05$
Statistics	$\chi^2 = 27.01$, df = 4, $p < 0.001$	$\chi^2 = 145.54$, df = 4, $p < 0.001$

Values are given in mean ± standard deviation. Different superscript letters within a column (i.e., treatment-wise) indicate significant differences (Dunn's post hoc test at 0.05% level).
