# Supplementary material for: Seasonal Variation in Flower Traits, Visitor Traits, and Reproductive Success of Solanum sisymbriifolium Lamarck (Solanaceae) in the Rarh Region of West Bengal, India
Source: Biology (Basel). 2025 Jul 16;14(7):865. doi: 10.3390/biology14070865 (PMC12292435; doi:10.3390/biology14070865)
Supplement: Supplementary file 1 [file biology-14-00865-s001.zip › 18. Table S5.pdf]

Table S5. Daytime-wise, visitor traits (abundance, richness, and diversity) of *Solanum sisymbriifolium* in West Bengal, India.

Visitor traits	Summer	Monsoon	Autumn	Late autumn	Winter	Spring
**Abundance**						
4:00–6:00 h	3.47^{b} ± 2.53	2.93^{b} ± 1.87	2.27^{b} ± 2.08	1.70^{bc} ± 1.62	0.60^{cd} ± 0.74	2.77^{b} ± 1.87
6:00–8:00 h	4.50^{a} ± 2.16	4.10^{a} ± 2.63	3.13^{a} ± 1.96	2.60^{a} ± 1.43	0.87^{bc} ± 0.78	3.77^{a} ± 1.96
8:00–10:00 h	3.20^{b} ± 1.63	2.80^{b} ± 2.01	2.43^{ab} ± 1.72	2.23^{ab} ± 1.50	1.40^{ab} ± 1.15	2.57^{b} ± 1.83
10:00–12:00 h	2.20^{c} ± 1.30	1.87^{c} ± 1.57	1.37^{c} ± 1.13	1.17^{cd} ± 0.91	1.77^{a} ± 1.46	1.70^{c} ± 1.42
12:00–14:00 h	1.87^{c} ± 1.07	1.50^{cd} ± 1.33	1.07^{cd} ± 1.08	0.90^{de} ± 0.99	0.80^{bc} ± 0.75	1.43^{cd} ± 1.22
14:00–16:00 h	0.93^{d} ± 0.87	0.83^{de} ± 0.91	0.57^{d} ± 0.77	0.47^{ef} ± 0.73	0.40^{d} ± 0.67	0.73^{de} ± 0.83
16:00–18:00 h	0.60^{d} ± 0.72	0.47^{e} ± 0.68	0.37^{d} ± 0.61	0.23^{f} ± 0.50	0.33^{d} ± 0.61	0.43^{e} ± 0.68
**Richness**						
4:00–6:00 h	0.56^{ab} ± 0.47	0.51^{ab} ± 0.45	0.51^{ab} ± 0.53	0.34^{ab} ± 0.47	0.34^{abc} ± 0.60	0.50^{abc} ± 0.48
6:00–8:00 h	0.79^{a} ± 0.39	0.74^{a} ± 0.50	0.68^{a} ± 0.54	0.56^{a} ± 0.50	0.39^{ab} ± 0.56	0.72^{a} ± 0.51
8:00–10:00 h	0.75^{a} ± 0.50	0.69^{a} ± 0.55	0.66^{a} ± 0.67	0.58^{a} ± 0.57	0.40^{a} ± 0.60	0.65^{ab} ± 0.56
10:00–12:00 h	0.60^{ab} ± 0.57	0.55^{ab} ± 0.57	0.34^{bc} ± 0.55	0.30^{ab} ± 0.57	0.14^{bc} ± 0.44	0.41^{bc} ± 0.50
12:00–14:00 h	0.54^{ab} ± 0.62	0.49^{ab} ± 0.60	0.27^{bc} ± 0.51	0.16^{ab} ± 0.42	0.13^{bc} ± 0.39	0.39^{bc} ± 0.58
14:00–16:00 h	0.25^{b} ± 0.53	0.25^{bc} ± 0.53	0.19^{c} ± 0.50	0.10^{b} ± 0.37	0.10^{c} ± 0.37	0.22^{cd} ± 0.51
16:00–18:00 h	0.14^{b} ± 0.44	0.10^{c} ± 0.37	0.10^{c} ± 0.37	0.05^{b} ± 0.26	0.10^{c} ± 0.37	0.10^{d} ± 0.37
**Diversity**						
4:00–6:00 h	0.49^{bc} ± 0.41	0.42^{bc} ± 0.36	0.38^{bc} ± 0.38	0.25^{ab} ± 0.34	0.19^{ab} ± 0.33	0.39^{bc} ± 0.36
6:00–8:00 h	0.68^{a} ± 0.33	0.63^{a} ± 0.40	0.51^{a} ± 0.40	0.40^{a} ± 0.34	0.26^{a} ± 0.36	0.57^{a} ± 0.39
8:00–10:00 h	0.55^{ab} ± 0.34	0.52^{ab} ± 0.40	0.43^{ab} ± 0.40	0.38^{a} ± 0.35	0.24^{a} ± 0.35	0.47^{ab} ± 0.38
10:00–12:00 h	0.39^{bc} ± 0.36	0.36^{bc} ± 0.35	0.19^{bc} ± 0.30	0.16^{bc} ± 0.29	0.07^{b} ± 0.20	0.28^{cd} ± 0.33
12:00–14:00 h	0.32^{c} ± 0.36	0.30^{cd} ± 0.35	0.15^{c} ± 0.28	0.09^{c} ± 0.23	0.07^{b} ± 0.21	0.23^{cd} ± 0.34
14:00–16:00 h	0.13^{d} ± 0.27	0.13^{de} ± 0.27	0.09^{d} ± 0.24	0.05^{c} ± 0.18	0.05^{b} ± 0.18	0.11^{de} ± 0.26
16:00–18:00 h	0.07^{d} ± 0.21	0.05^{e} ± 0.18	0.05^{d} ± 0.18	0.02^{c} ± 0.13	0.05^{b} ± 0.18	0.05^{e} ± 0.18
**Statistical analysis**	Abundance: χ2 = 85.73, ***; Richness: χ2 = 34.99, ***; Diversity: χ2 = 51.01, ***	Abundance: χ2 = 63.31, ***; Richness: χ2 = 33.46, ***; Diversity: χ2 = 45.22, ***	Abundance: χ2 = 63.26, ***; Richness: χ2 = 34.04, ***; Diversity: χ2 = 41.20, ***	Abundance: χ2 = 70.10, ***; Richness: χ2 = 39.69, ***; Diversity: χ2 = 42.82, ***	Abundance: χ2 = 40.62, ***; Richness: χ2 = 18.38, **; Diversity: χ2 = 19.56, ***	Abundance: χ2 = 72.15, ***; Richness: χ2 = 33.15, ***; Diversity: χ2 = 42.61, ***

Values are given in mean ± standard deviation. Statistical analysis: different superscript letters within a column indicate significant differences (Dunn's post hoc test at 0.05% level); Kruskal-Wallis H test: df = 6, p<0.001 (***), p<0.01 (**), p<0.05 (*).
