# Supplementary material for: Seasonal Variation in Flower Traits, Visitor Traits, and Reproductive Success of Solanum sisymbriifolium Lamarck (Solanaceae) in the Rarh Region of West Bengal, India
Source: Biology (Basel). 2025 Jul 16;14(7):865. doi: 10.3390/biology14070865 (PMC12292435; doi:10.3390/biology14070865)
Supplement: Supplementary file 1 [file biology-14-00865-s001.zip › 14. Table S1.pdf]

Table S1. The methodologies for the microscopic study of flower parts and pollen grains.

Phase	Different steps
Preparation of flower parts (e.g., sepals, petals, stigma, and ovary) for scanning electron microscope (SEM) study	1. A small piece of a flower part (e.g., sepal, petal, stigma, and ovary) was taken in a vial.<br>2. Flower parts were fixed in a 3% glutaraldehyde solution.<br>3. After discarding the glutaraldehyde solution, the sample was gradually dehydrated using ethanol at increasing concentrations (50%, 70%, and 100%).<br>4. Then, the sample was air-dried.
SEM study of flower parts	1. The prepared flower sample was affixed to metal stubs using sticky carbon tape to enhance conductivity.<br>2. To mitigate charge buildup on the specimen surface, a 10 nm layer of gold nanoparticles was applied as a conductive coating using the Q150R ES sputter coater (Quorum Technologies, UK).<br>3. The samples were examined using a field emission scanning electron microscope (FE-SEM, GeminiSEM 450, Gemini 2 column) from Zeiss, Gemini, India. Surface images of the sample were captured under high-vacuum conditions at magnifications ranging from 1x to 40x. Imaging was performed using a secondary electron detector with an acceleration voltage of 5.0 kV.
Processing of the pollen sample through the acetolysis method	1. Pollen grains were collected from flower anthers.<br>2. Pollen grains were stored in a vial with 70% ethanol.<br>3. Centrifuged at 3000 rpm for 5 min and decanted the supernatant.<br>4. Added glacial acetic acid to the pollen pellet and shook.<br>5. Centrifuged at 3000 rpm for 5 min and decanted the supernatant.<br>6. Added acetolysis mixture (anhydrous acetic acid and conc. H_2SO_4 in a 9:1 ratio) to the pollen pellet, shook, and placed in a water bath at 100°C for 3 min.<br>7. Centrifuged at 3000 rpm for 5 min and decanted the supernatant.<br>8. Added distilled water, shook, and rinsed, and centrifuged at 3000 rpm for 5 min, and decanted the supernatant.
Light microscopic study of pollen grains	1. Pollen sediment was taken on a glass slide with glycerine jelly.<br>2. The jelly-containing pollen was warmed to melt it and covered with a coverslip.<br>3. We sealed the coverslip by using nail polish.<br>4. Then, we observed the pollens using a light microscope (Primo Star, Zeiss) and took pollen microphotographs.
Processing of the pollen sample for the SEM study	1. Pollen grains were collected from flower anthers.<br>2. Pollen grains were fixed in a 3% glutaraldehyde solution.<br>3. Then, the glutaraldehyde was discarded by centrifugation.<br>4. Then, the pollen grains were gradually dehydrated using ethanol with increasing concentrations.<br>5. After centrifugation, pollen grains were air-dried.
SEM study of pollen grains	1. The pollen samples were affixed to metal stubs using sticky carbon tape to enhance conductivity.<br>2. Then, we followed a similar methodology to the SEM study of flower parts.
SEM study of the visitor's body	1. The dried whole insects and body parts (e.g., legs, head, thorax, and abdomen) were affixed to metal stubs using sticky carbon tape to enhance conductivity.<br>2. Then, we followed a similar methodology to the SEM study of flower parts.
