# Supplementary material for: Seasonal Variation in Flower Traits, Visitor Traits, and Reproductive Success of Solanum sisymbriifolium Lamarck (Solanaceae) in the Rarh Region of West Bengal, India
Source: Biology (Basel). 2025 Jul 16;14(7):865. doi: 10.3390/biology14070865 (PMC12292435; doi:10.3390/biology14070865)
Supplement: Supplementary file 1 [file biology-14-00865-s001.zip › 15. Table S2.pdf]

Table S2. Methodologies for determining relative abundance, richness and diversity of flower visitors.

Parameter	Methodology
Relative abundance (RA)	$$\text{RA}\ (\%) = \frac{\text{n}i}{\text{N}} \times 100$$ The ni is the number of individuals recorded for an insect species *i*, and N is the total number of individuals recorded for all insect species.
Visitors' richness [the index (D) of Margalef (1958)]	$$\text{D} = \frac{\text{S} - 1}{ln\,\text{N}}$$ S is the number of flower-visiting species, and N is the total number of individuals observed. The natural logarithm, denoted as *ln*. The index D was calculated for each sample. Each sample corresponded to a single survey, representing a 5-minute observation on an individual plant.
Visitors' diversity [the diversity index (*H'*) of Shannon-Weaver (1949)]	$$H' = -\sum_{i}^{n}(\text{pi}.\,ln\,\text{pi})$$ Here, pi represents the proportion of each visitor species within the sample (pi = ni/N, where ni is the number of individuals recorded for species *i*, and N is the total number of individuals recorded in the sample).
